# Facial Diplegia: A Rare, Atypical Variant of Guillain-Barré Syndrome and Ad26.COV2.S Vaccine

**DOI:** 10.7759/cureus.16612

**Published:** 2021-07-25

**Authors:** Esha Jain, Krunal Pandav, Pratima Regmi, George Michel, Ida Altshuler

**Affiliations:** 1 Medicine, American University of Antigua, St. John's, ATG; 2 Division of Research & Academic Affairs, Larkin Community Hospital, South Miami, USA; 3 Biology, College of Staten Island, New York, USA; 4 Internal Medicine, Larkin Community Hospital, South Miami, USA; 5 Neurology, Richmond University Medical Center, New York, USA

**Keywords:** guillain barre’s syndrome (gbs), facial diplegia, johnson and johnson vaccine, ad26.cov2.s vaccine, facial nerve palsy

## Abstract

This potentially life-threatening disease poses an interesting perspective on adverse events that can occur or can be exacerbated following the Ad26.COV2.S (Johnson & Johnson) vaccine. The authors report findings in a 65-year-old female patient who experienced facial diplegia, an atypical variant of Guillain-Barré syndrome, two weeks after receiving the Ad26.COV2.S vaccine against coronavirus disease 2019. Post-approval pharmacovigilance of each vaccine helps better understand the long-term outcomes, and reporting adverse events is crucial for advancements in medical knowledge.

## Introduction

Reporting adverse events is crucial since post-approval pharmacovigilance of the vaccinations helps researchers and clinicians better understand the long-term outcomes. Thereby, the authors report a unique case of a 65-year-old female patient who experienced facial diplegia, two weeks after receiving the Ad26.COV2.S vaccine. Guillain-Barré syndrome (GBS) can be defined as symmetrical, ascending motor weakness along with areflexia and cerebrospinal fluid (CSF) findings yielding albuminocytologic dissociation. Facial diplegia is a rare [1] and an atypical variant of GBS [2], which can be easily misdiagnosed [1]. To our knowledge, this is the first report of the facial diplegia post-coronavirus disease 2019 (COVID-19) vaccine. We present this case with an aim to increase awareness of the condition.

## Case presentation

A 65-year-old Caucasian female who received the Ad26.COV2.S vaccine presented to the emergency department (ED) in Staten Island, New York. She had a past medical history of hypertension, diabetes mellitus type 2, and hyperlipidemia and reported allergies to ciprofloxacin, codeine, penicillin, and egg whites. The patient endorsed a headache in the frontal and periorbital regions for four days, along with left neck and shoulder pain from day 15 post-vaccination. Simultaneously, she experienced ageusia and hyposalivation. On day 19 post-vaccination, the patient woke up with dysarthria, dysphagia, dysphasia, and bilateral facial weakness, which prompted the patient to visit the ED of the Richmond University Medical Center the following day. Neurological assessment upon arrival showed that the patients' Glasgow Coma Scale was 15/15, and the patient had significant dysarthria. Gag reflex was weak and there was a bilateral loss of power in the distribution of the facial nerve. The remainder of the central and peripheral neurological examination was normal. Chest X-ray and computed tomography (CT) of the head were negative. Magnetic resonance imaging (MRI) of the head demonstrated no acute infarct, hydrocephalus, acute intracranial hemorrhage, or mass effect. CT angiography of the head and neck demonstrated less than 50% stenosis in left and right internal carotid arteries.

A stroke code was activated on patient arrival to the ED, which was ruled out by the neurology team. Due to the bilateral cranial nerve (CN) VII lower motor neuron lesions, the patient was promptly admitted to the intensive care unit. A lumbar puncture was performed to rule out the Miller Fisher variant of GBS. Results of CSF showed protein of 302 mg/mL, and absence of white blood cell yielding albuminocytologic dissociation with IgA level of 163 mg/dL. Following diligent clinical and physical assessment, and analyzing CSF results via lumbar puncture, empiric treatment was initiated for GBS, and the patient was diagnosed with facial diplegia, a rare variant of GBS [2]. The patient was administered intravenous immunoglobulin (IVIG) (0.4 g/kg) for 10 days followed by 10 sessions of plasmapheresis. She was provided with supplemental oxygen and close monitoring for potential diaphragmatic paralysis.

**Figure 1:**
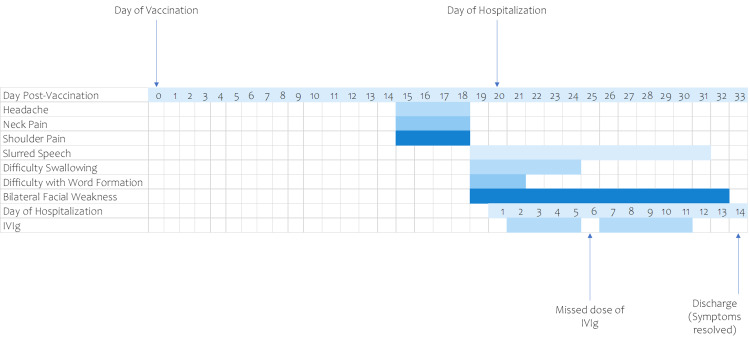
Clinical course from day of vaccination to discharge from hospital

On day 3 of hospitalization, the patient was awake, alert, oriented, and able to follow commands. She denied any respiratory distress; however, slurred speech with mild dysarthria was noted. On examination of CN II to XII, the pupils reacted light bilaterally. Extraocular muscles were intact bilaterally, and nystagmus/diplopia was absent. When asked to close her eyes, there was a bilateral loss of power in the orbicularis oculi muscles. There was a significant CN VII lower motor neuron lesion bilaterally. The patient exemplified bilateral upper and lower facial motor weakness and decreased gag reflex. The tongue and uvula were in the midline, and there was a normal, symmetric movement of the soft palate. Motor examination showed a normal muscle tone throughout all four extremities. Power was 4/5 in the left lower extremity. Deep tendon reflexes were 0 at the left upper extremity, 1+ at the right upper extremity, bilateral knees, and left ankle were 0, and the right ankle had 2+ reflex present. Sensory examination was normal, and toes were down-going bilaterally. Acetylcholine receptor binding and modulating antibodies were negative, and anti-GQ1b antibodies <1:100 (normal titer is <1/200). On day 9 of hospitalization, the patient showed notable improvement in her speech. CN examination showed an improvement in the power in the orbicularis oculi muscles. There was notable bilateral CN VII lower motor neuron lesion, bilateral upper and lower facial weakness, and decreased gag reflex. Muscle tone was normal in all four extremities; however, power in the left lower extremity was 4/5. Deep tendon reflexes of the left upper extremity and right upper extremity were 0 and 1+, respectively. Furthermore, that of bilateral knees, left ankle, and right ankle were 1+, 0, and 2+, respectively. Sensory examination was intact. The remainder of the CN exam was the same as day 3. On day 12 of hospitalization, the patient's speech was slightly slurred with mild dysarthria, and the patient was able to close her eyes and keep them closed, which she was unable to do before. Nasolabial folds were slightly deeper. Bilateral CN VII lower motor neuron lesion signs were still present with bilateral upper and lower facial weakness and decreased gag reflex. On day 14, the patient was discharged and outpatient follow-up with a neurologist was recommended. Treatment with IVIG and plasmapheresis was observed to be successful with the patient being able to return to her normal baseline health at the time of discharge as seen in Figure 1. This event was reported to the Vaccine Adverse Event Reporting System.

.

## Discussion

GBS is a rare but severe autoimmune condition that presents with symmetrical polyradiculoneuropathy and has an annual incidence of around 0.6-4.0 per 100,000 [3]. An antecedent infection before symptoms of GBS is seen in around 70% of the GBS patients [4]. *Campylobacter jejuni*, cytomegalovirus, Epstein-Barr virus, human immunodeficiency virus (HIV), and Zika virus have been associated with GBS [3]. Recently, an association of GBS with severe acute respiratory syndrome coronavirus 2 was also shown in a case report [3]. Studies have also linked medications, surgeries, and vaccinations with GBS. A well-known example is an increase in GBS incidence after flu vaccination against the influenza A/H1N1 antigen, documented in 1976. However, in later years, one GBS case per one million vaccines has been described in the surveillance data [4]. In a recent study, two participants of the Ad26.COV2.S vaccine trial developed GBS. Out of the two participants, one received the vaccine, and the other received a placebo. Since the participant who received the vaccine also developed GBS, it was not proved that the vaccine directly caused GBS in that patient [5].

**Figure 2:**
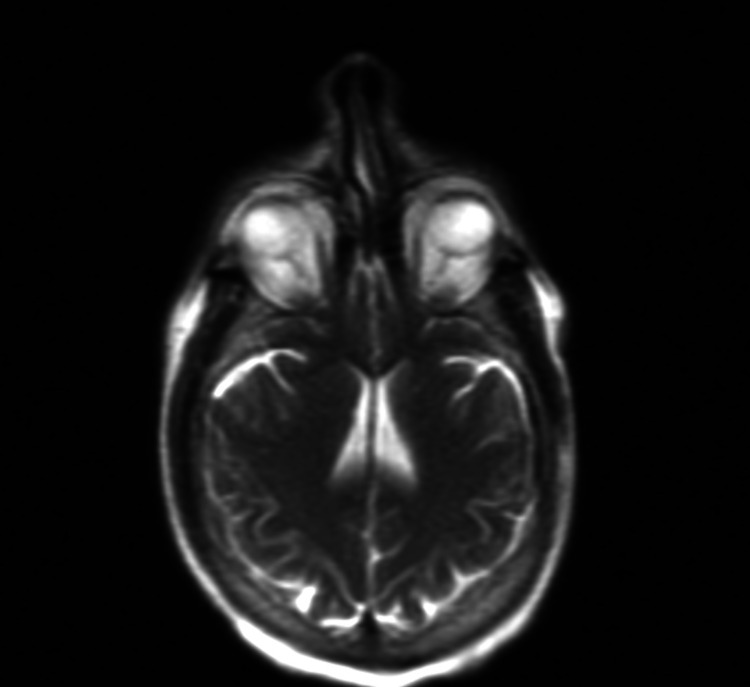
Axial plane of brain MRI with contrast MRI, magnetic resonance imaging.

Facial diplegia is an atypical and relatively rare variant of GBS that occurs in less than 1% of the patients diagnosed with GBS [6]. Bilateral facial paralysis and paresthesia are commonly seen in facial diplegia patients [7]. Multiple pathologies can lead to bilateral facial diplegia, including Bell's palsy, sarcoidosis, Lyme disease, leprosy, Bickerstaff brainstem encephalitis (BBE), brainstem stroke, herpes zoster, and HIV [8]. BBE and GBS closely resemble each other's clinical presentation. It is crucial for physicians to distinguish between BBE and the facial diplegia variant of GBS in order to provide appropriate management. Our patient had negative titers of IgG anti-GQ1b antibody, which is commonly found in patients presenting with BBE or Fisher syndrome. Furthermore, patients with BBE, presenting without IgG anti-GQ1b antibodies, often had abnormal findings on brain MRI [9]. In comparison to the case we presented, our patient showed normal findings on brain MRI, as seen in Figure 2, effectively ruling out BBE.

This patient denied a family history of any variant of GBS, and there were no other identifiable triggering factors. The symptoms such as numbness, tingling, weakness, and paralysis result from autoimmune destruction of the nerves of the peripheral nervous system [6]. When a patient presents with bilateral facial paralysis, diagnosis depends on their clinical presentation with emphasis on their history [6]. A detailed history should include timing of onset, prior history of facial paralysis, recent upper respiratory tract infection, change in taste, facial numbness, and so on. Thorough physical examination should be conducted with emphasis on neurological assessment focusing on the head and neck regions. The most common cause of facial diplegia due to infection is Lyme disease, which was ruled out in our patient via serum test. Ruling this out with an immunologic assay using antibody titers is crucial [6].

This patient demonstrated one of the many adverse events that may arise after any vaccination. With the recent introduction of vaccines against COVID-19, potential adverse events are still being discovered and investigated. Vaccination is imminent to control the ongoing pandemic situation. In addition, post-approval pharmacovigilance of each vaccine is crucial to help clinicians better understand such events, making it necessary to report the adverse events.

## Conclusions

To our knowledge, this is the first report of facial diplegia post-COVID-19 vaccination. The purpose of this article is to bring awareness to the facial diplegia variant of GBS and the importance of reporting the potential adverse reactions post-COVID-19 vaccination. Prompt management with IVIG should be initiated if facial diplegia is diagnosed. Adverse events after vaccination must be reported as they need to be further investigated, considering the fact that vaccination is needed to control the ongoing pandemic situation.
